# Revisiting ovarian cancer microenvironment: a friend or a foe?

**DOI:** 10.1007/s13238-017-0466-7

**Published:** 2017-09-19

**Authors:** Boyi Zhang, Fei Chen, Qixia Xu, Liu Han, Jiaqian Xu, Libin Gao, Xiaochen Sun, Yiwen Li, Yan Li, Min Qian, Yu Sun

**Affiliations:** 1 Key Laboratory of Stem Cell Biology, Institute of Health Sciences, Shanghai Institutes for Biological Sciences, Chinese Academy of Sciences and Shanghai Jiao Tong University School of Medicine, University of Chinese Academy of Sciences, Shanghai, 200031 China; 2 Institute of Health Sciences, Shanghai Jiao Tong University, School of Medicine and Shanghai Institutes for Biological Sciences, Chinese Academy of Sciences, Shanghai, 200031 China; 3 Department of Medicine and VAPSHCS, University of Washington, Seattle, WA 98195 USA

**Keywords:** ovarian cancer, stromal cells, tumor microenvironment, therapeutic resistance, ectopic metastasis, combinational treatment, patient stratification

## Abstract

Development of ovarian cancer involves the co-evolution of neoplastic cells together with the adjacent microenvironment. Steps of malignant progression including primary tumor outgrowth, therapeutic resistance, and distant metastasis are not determined solely by genetic alterations in ovarian cancer cells, but considerably shaped by the fitness advantage conferred by benign components in the ovarian stroma. As the dynamic cancer topography varies drastically during disease progression, heterologous cell types within the tumor microenvironment (TME) can actively determine the pathological track of ovarian cancer. Resembling many other solid tumor types, ovarian malignancy is nurtured by a TME whose dark side may have been overlooked, rather than overestimated. Further, harnessing breakthrough and targeting cures in human ovarian cancer requires insightful understanding of the merits and drawbacks of current treatment modalities, which mainly target transformed cells. Thus, designing novel and precise strategies that both eliminate cancer cells and manipulate the TME is increasingly recognized as a rational avenue to improve therapeutic outcome and prevent disease deterioration of ovarian cancer patients.

## Introduction

Ovarian cancer represents the 5th leading cause of cancer-related death in women, which comprises a genetically and histologically broad range of tumors including those of epithelial, germ cell, and sex cord-stromal origin (Karnezis et al., 2017). One of the main facts that cause difficulty in understanding the biology and evolution of ovarian cancer is that most neoplastic cells are phenotypically not similar to their normal counterparts (Aiello et al., 2017). For high-grade serous carcinoma (HGSC), the most common ovarian malignancy usually diagnosed at an advanced stage with high mortality, no credible precursor lesion was histologically identified until 15 years ago, with the majority of mucinous ovarian cancers being in nature metastases from other organs including nearby sites such as the pancreas (Young, 2006).

Indeed, a wide variety of cancers that are not derived from normal ovarian cell types including primary ovarian neoplasia and metastatic cancers from the breast, lung, and the gastrointestinal (GI) tract-exist as major ovarian masses (Worzfeld et al., 2017). In contrast to the ovary which is generally understood as a fertile environment supportive of precancerous and cancerous lesions, the fallopian tube acts as a presumably inhospitable microenvironment for growth of both primary and metastatic tumors (McDaniel et al., 2015; Rabban et al., 2015). It is reasonable to speculate that the fallopian tube has evolved a tumor-suppressive microenvironment to minimize the chance of ectopic pregnancy, a condition essentially lethal until the era of surgery (Karnezis et al., 2017). Of note, the size of most ovarian malignancies, including primary tumors and non-gynaecological metastases, is significantly larger than the normal ovary and among the largest malignancies in the body. Although tumor stroma is usually generated *de novo,* the pathophysiological role of the stroma in ovarian neoplastic growth remains unclear. Earlier studies indicated that ovarian tumor stroma display both endo-crinological and morphological features of normal adult ovary, while ovarian metastases from GI tract malignancies have stroma similar to neither that of the primary GI tract tumors nor extra-ovarian metastases by containing luteinized, steroidogenic ovarian stromal cells (Scully and Richardson, 1961). Thus, the stroma of ovarian tumors histologically resembles that of normal adult ovary, and can likely provide a microenvironment that promotes the development of primary and metastatic tumors. Beyond this, comparative profiling of the transcriptomics and proteomics of ovarian tumor stroma by contrasting with those of alternative tumor types such as primary breast cancer versus ovarian metastases, may disclose stimulatory factors correlated with the preferential growth of ovary cancer as potential therapeutic targets.

Although it is well accepted that epithelial ovarian cancer cells are responsive to steroid hormone stimulation, new studies have presented the clues that the ovarian stroma may also have an active role in this process. For instance, ovarian stroma immediately adjacent to the tumor foci can express markers associated with sex-steroid differentiation and steroidogenesis (calretinin, inhibin, and steroidogenic factor 1), alongside steroid enzymes (CYP17, CYP19, HSD17β1, and AKR1C3), while the epithelium expresses corresponding hormone receptors (Blanco et al., 2017). Thus, the epithelium-surrounding stroma in the ovary is activated to elaborate biologically relevant hormones which may enhance incontrollable neoplastic growth, although the precise mechanisms underlying these processes await further investigation. Specifically, isoform-specific alterations of Akt, the serine-threonine kinase whose 3 isoforms are encoded by distinct genes and frequently overexpressed in numerous cancers, were recently found to have divergent effects in ovarian cancer cells and the nearby microenvironment (Linnerth-Petrik et al., 2016). Ablation of Akt1 in the TME generated an inhibitory effect on tumor size, without significant change in animal survival, while elimination of Akt2 or Akt3 resulted in increased tumor size, metastasis, and decreased survival time (Linnerth-Petrik et al., 2016). Although it is increasingly evident that stromal components have significant clinical implications in ovarian cancer development, recent findings uncovered an even stronger impact orchestrated by diverse cell types that may predict overall and progression-free survival of HGSC (Heindl et al., 2016). Beyond, quantitative histology-based assessments can further enable appropriate selection of patients who are in urgent need of specific therapeutic strategies including combinatorial treatments that target the heterogeneous TME (Heindl et al., 2016).

A typical TME comprises diverse non-cancerous cell lineages, including stromal fibroblasts, infiltrating leukocytes, adipocytes, neuroendocrine cells, endothelial cells, and pericytes (Chen et al., 2015). According to the specific stage of disease progression and the particular organ type, TME cells can play tumor-promoting or tumor-suppressing roles, partially depending on the adjacent cancer cells that have co-evolved. Importantly, some of the functional mechanisms through which the TME influences pathological progression are also “co-opted” to drive ectopic metastasis and therapeutic resistance in clinical settings (Klemm and Joyce, 2015).

One of the main properties that distinguish ovarian cancer from other solid tumors is the specific TME within the ovary. As ovarian cancer is a peritoneal malignancy, cancer cell dissemination is partially dependent on the peritoneal fluid as a carrier (Kipps et al., 2013). In such a case, transcoelomic dissemination is a major route of cancer cell adhesion to the omentum and serous membranes that line the peritoneal organs, generating metastatic lesions in the peritoneal cavity instead of invading through the lamina propria (Lengyel, 2010). The peritoneal environment is frequently formed by the effusion accumulating in the peritoneal cavity, which presents as large volumes of ascites (Mikula-Pietrasik et al., 2016). Typically, the ascites comprises detached cancer cells, numerous soluble factors, extracellular vesicles (EVs), various types of immune cells including T cells and tumor-associated macrophages (TAMs), as well as many other host cell subpopulations, together favoring cancer cell proliferation, chemoresistance, and metastasis (Pogge von Strandmann et al., 2017). Distinct from most other human malignancies, metastases at distant sites are often confined to late stages of ovarian cancer, and the most serious problem for HGSC patients is recurrent and aggressive growth of metastatic lesions within the peritoneal cavity (Pogge von Strandmann et al., 2017). The second feature of ovarian cancer is the special relevance of the omentum, a physical structure composed of connective and fatty tissue that covers the ventral surface of the intestines. Specifically, the omentum is often the preferred site for ovarian cancer metastases and plays a key role in disease progression (Lengyel, 2010).

## CANCER-ASSOCIATED FIBROBLASTS

Pathological development of ovarian cancer, from cell transformation to local tissue invasion and distant metastatic dissemination, relies on mutual communication between epithelial ovarian cancer (EOC) cells and their adjacent stromal microenvironment. An appropriate understanding of the bidirectional interaction of early EOC cells with activated stromal cells helps identify novel diagnostic stromal markers and molecular targets for clinical therapy. The stroma comprises up to 50% of the advanced ovarian tumor mass, wherein cancer-associated fibroblasts (CAFs, sharing many common features with myofibroblasts) represent a major cell subpopulation in the local TME (Labiche et al., 2010). By producing secretory factors such as hepatocyte growth factor (HGF), CAFs remarkably decrease sensitivity of cancer cells to various anticancer agents (Straussman et al., 2012; Wilson et al., 2012). Moreover, CAFs alter the tumor physical properties via excessive deposition and aberrant remodeling of the extracellular matrix (ECM), thus enhancing formation of an interstitial barrier that blocks efficient drug delivery (Sun, 2015). Specifically, targeting CAFs increases bioavailability of chemotherapeutic agents including doxorubicin and gemcitabine, while enabling immunological surveillance and tumor destruction through a process that engages interferon-gamma (IFNγ) and tumor necrosis factor alpha (TNF-α) (Kraman et al., 2010; Olive et al., 2009).

The range of biological mechanisms employed by CAFs to mediate therapeutic resistance was expanded by a recent study, which identified a novel role of CAFs in minimizing cisplatin levels within ovarian cancer cells upon chemotherapy (Wang et al., 2016). Specifically, CAF-derived glutathione and cysteine contribute to treatment resistance, which can be abolished by CD8(+) T cells by altering metabolism of these molecules in CAFs (Wang et al., 2016). CD8(+) T-cell-released IFNγ regulates glutathione and cysteine levels via upregulation of γ-glutamyl-transferases and transcriptional inhibition of system xc(−) cystine and glutamate antiporter through the JAK/STAT1 axis. Importantly, the presence of stromal CAFs and CD8(+) T cells is negatively and positively correlated with the survival of ovarian cancer patients, respectively, thus capitalizing the interaction between chemotherapy and immunotherapy holds significant potential to improve treatment outcomes of ovarian cancer patients (Wang et al., 2016). As immunotherapy is emerging as a mainstay of anticancer strategies in several malignancy types including ovarian cancer, novel avenues for therapeutically targeting the pro-tumorigenic CAFs through modulation of CD8^+^ T cells are highly inspiring to both scientific and clinical communities. Although some technical issues still remain regarding how to effectively harness the TME in therapeutic settings, future studies that explore the interplay between the CAF populations and adaptive immune cells hold significant potential to provide updated approaches to integrate T cell therapy and/or fibroblast depletion, thereby enhancing the overall efficacy of DNA damaging chemotherapies.

During carcinogenesis, CAFs exhibit increased production and deposition of ECM components such as collagens, and are phenotypically distinguished from normal fibroblasts with enhanced expression of two CAF markers including α smooth muscle actin (α-SMA) and fibroblast activation protein (FAP) (Schauer et al., 2011). CAFs can control epithelial cell differentiation through the secretion of multiple soluble factors, while the paracrine signaling between EOC cells and CAFs results in the release of growth, migratory and invasive signals that substantially accelerate disease progression. For example, cytokines present within medium conditioned from ES-2, an ovarian clear cell carcinoma cell line, induced transcription of urokinase-type plasminogen activator (uPA) mRNA in fibroblast cells, eventually generating enhanced urokinase implicated in cancer invasion and migration (Noskova et al., 2009). Moreover, a premetastatic niche was created in the omentum via activation and proliferation of normal fibroblasts, which were subject to stimulation by cancer cell-derived transforming growth factor β1 (TGF-β1) (Cai et al., 2012). Establishment of such a niche promoted tumor invasion at peritoneal surfaces, mainly through fibroblast release of HGF and matrix metalloproteinase 2 (MMP2). A83-01, an inhibitor of the TGF-β type I receptor, abrogated TGF-β1 signaling and proliferation of normal fibroblasts, accompanied by α-SMA and MMP2 expression in tumors that contain SKOV3 cells and fibroblasts. Data from another study suggest that CAFs are present in higher abundance in advanced cancer stages, and are associated with increased density of lymphatic vessel and microvessel as well as enhanced metastasis to lymph node and omentum (Zhang et al., 2011). Specifically, CAFs isolated from ovarian cancer patients induced more significant cancer cell migration than fibroblasts from normal ovarian tissues.

Although increasing evidence suggests that selectively targeting TME cells could be a feasible approach to harness the tumor-stroma communication, identification of stroma-specific targets to make cancer cells vulnerable to therapeutic agents remains an intriguing but challenging task. To fill such a blank, a new study discovered that CAFs boost glutamine production by utilizing carbon and nitrogen from atypical nutrient sources to sustain cancer cell growth in the absence of sufficient glutamine in the TME (Yang et al., 2016). Data from an ovarian cancer orthotopic mouse model even demonstrated that co-targeting CAF glutamine synthetase and cancer cell glutaminase can disrupt such a metabolic crosstalk, inducing pronounced disease regression (Yang et al., 2016). Albeit partially uncovering the details of ovarian TME complexity, this pilot strategy is likely to synergize with multiple traditional therapies that target autonomous mechanisms of cancer cells, and holds the promise to contribute ground-breaking inputs to our ever increasing anticancer arsenal in clinics (Tajan and Vousden, 2016).

## MESENCHYMAL STEM CELLS

Mesenchymal stem cells (MSCs) represent an active stromal cell subpopulation that is recruited to the TME, with prominent multipotency that allows differentiation into various cell types. In certain cases, the recruitment may be partly induced by LL-37 (leucine, leucine-37), a proinflammatory peptide of human cationic antimicrobial protein 18, and other migratory signals (Coffelt et al., 2009). Human bone marrow-derived MSCs can differentiate into CAFs, which produce soluble pro-tumorigenic factors such as interleukin 6 (IL-6) to enhance tumor growth in an EOC xenograft model (Spaeth et al., 2009). Combining cancer cells and cancer-associated MSCs *in vivo* and *in vitro* causes activation of the bone morphogenetic protein (BMP) signaling network, which plays important roles in cancer progression (McLean et al., 2011).

Co-injection of ovarian MSCs, which secret a high level (>2,500 pg/mL) of IL-6, with SKOV3 cells enhanced tumorigenesis, sphere and colony formation in non-obese diabetic-severe combined immunodeficiency (NOD-SCID) mice, while administration of an IL-6 receptor blocking antibody minimized these malignant behaviors (Ding et al., 2016). Interestingly, cancer exosome-treated MSCs have elevated α-SMA expression, a change that indicates an activated fibroblast phenotype, alongside increased synthesis of tumor-promoting cytokines including stromal cell-derived factor-1 (SDF-1) and TGF-β (Cho et al., 2011). Thus, ovarian cancer-derived exosomes can contribute to the generation of CAFs differentiated from MSCs in tumor stroma.

Development of enhanced chemoresistance to standard clinical therapies is not uncommon in cancer patients, frequently allowing cancer cells to acquire a “cancer stem cell (CSC)-like” phenotype. This phenotypic change is usually accompanied by an epithelial-mesenchymal transition (EMT), the phenotypic switch mostly implicated in cancer metastasis. For instance, the metastatic cell line OVCA433 exhibits upregulated expression of EMT and stem cells markers (including CD44, α2 integrin subunit, CD117, CD133, EpCAM, Nanog, and Oct-4), and enhanced activation of extracellular regulated kinase 2 (ERK2) signaling upon treatment with cisplatin (Latifi et al., 2011). To the contrary, ERK2 signaling blockage by a MEK inhibitor U012 diminished expression of EMT and CSC markers, implying the potential of targeting this pathway to reduce residual tumor burden, a common cause of ovarian cancer recurrence.

Both proliferation and invasion of human EOC cells are remarkably enhanced upon co-culture with omental adipose-derived MSCs (O-ADSCs) *in vitro*, and a recent study revealed a global increase in protein expression in the EOC cells treated with conditioned media (CM) from O-ADSC (Zhang et al., 2017). Specifically, nine proteins were identified with differential expression after CM treatment, which are linked to carcinogenesis, apoptosis and migration of cancer cells, suggest that O-ADSCs alter the proteomic profile of EOC cells via paracrine mechanism in favor of EOC progression (Zhang et al., 2017).

On the other hand, MSCs can be exploited as vehicles in cancer therapy, particularly when transduced with recombinant adenoviruses such as those encoding endostatin, an angiogenesis inhibitor (Jiang et al., 2010). Upon induction by SKOV3 cells *in vitro*, these transduced MSCs display increased capacity of migration, and can in turn generate antiproliferative effects on cancer via endostatin secretion. Furthermore, MSCs derived from human umbilical blood mononuclear cells can act as delivery vehicles for IL-21 administration to nude mice xenografted with ovarian cancer cells, a process that boosts antitcancer immunity in the murine models (Hu et al., 2011).

A new study explored the possibility of using engineered MSCs derived from adipose tissues to express either herpes simplex virus thymidine kinase (HSVtk-MSC), an enzyme that phosphorylates ganciclovir (GCV) to its toxic metabolites, or yeast fused cytosine deaminase::uracil phosphoribosyltransferase (CD::UPRT-MSC), another enzyme that converts 5-fluorocytosine (5-FC) to highly toxic 5-fluorouracil (5-FU) (Toro et al., 2016). As a result, significantly decreased tumor volumes of subcutaneous xenografts of EOC cells in nude mice and prolonged tumor-free survival in animals bearing these cancer cells after CD::UPRT-MSC/5-FC treatment were achieved. Taken together, these studies provide supporting evidence for the potential utility of MSCs as gene delivery vehicles that can be incorporated into future therapeutic strategies. Nevertheless, the possibility for MSCs to undergo either malignant transformation or differentiation into pro-tumorigenic fibroblasts can limit their roles in multiple clinical settings, thus future research is eagerly awaited to overcome these barriers and translate the medical value of MSCs in clinics.

## OVARY-ASSOCIATED ADIPOCYTES

For years, adipocytes are considered as energy producing and storing residents of fat tissue in the TME (Miranda and Ahmed, 2017). However, recent studies indicate that adipocytes may have other pathophysiological functions, and their interactions with cancer cells have been reported in breast, ovarian, colon, and gastric malignancies (Nieman et al., 2011). So far, tumor-promoting effects of adipocytes are linked to their secretion of adipokines, hormones, and growth factors including matrix metalloproteinase 11 (MMP11), IL-6, IL-1β into the surrounding TME, which collectively enhance the migration and invasion capacity of cancer cells (Dirat et al., 2011). In addition, upon co-culture with breast cancer cells, adipocytes exhibit an activated phenotype characterized by enhanced production of proteases and cytokines including IL-6 and IL-1β, as well as delipidation and a loss of adipocyte-associated markers. Further, peritumoral adipocytes exhibit an altered phenotype with specific biological features that are sufficient to allow these cells to be named cancer-associated adipocytes (CAA) (Dirat et al., 2011). Normal adipocytes stimulate the migration and invasion of cancer cells that are estrogen receptor (ER)-negative, a process mediated via a cytoskeletal element cofilin-1 and increased IL-6 secretion in adipocytes (Walter et al., 2009). Beyond the adipose tissue, pre-adipocytes also reside in the bone marrow and stromal compartments of other organs such as skin (Schmidt and Horsley, 2013). Interactions between mesenchymal stroma and the underlying adipose tissue allow generation of MSCs and cytokines, each frequently triggering stromal cell senescence and increasing therapeutic resistance (Ackerman and Simon, 2014; Sheng and Mittelman, 2014; Tchkonia et al., 2010). A new study exploring the mechanism of resistance to mitochondria-initiated apoptosis in EOC cells uncovered that adipocyte-induced upregulation of Bcl_xl_ in EOC cells was correlated with acquired chemoresistance, illustrating a novel pathway that allows the TME to modulate apoptosis-associated protein expression and confers chemoresistance on malignant cells (Cardenas et al., 2017).

A study using fluorescence technique to show the preferential migration of ovarian cancer cells to the mouse omentum revealed that cancer cell migratory behavior can be mediated by adipokines secreted by omentum-associated adipocytes, including chemokine C-C motif ligand 2 (CCL2), IL-6, IL-8, tissue inhibitor of metalloproteinase 1 (TIMP1), and adiponectin (Nieman et al., 2011). Of note, co-culture of adipocytes and ovarian cancer cells induced adipocyte-specific lipolysis, allowing the transfer of free fatty acids to cancer cells which in turn accelerated tumor growth through energy generation via β-oxidation. In addition, adipocyte-derived hormones such as leptin are associated with increased proliferation of ER-positive ovarian cancer cells, while ERα can be transcriptionally activated through the signal transducer and activator of transcription-3 (STAT3) signaling pathway, suggesting that both ER status and growth promoting properties of adipocytes need to be considered in obese ovarian cancer patients (Choi et al., 2011).

As adipokines can control multiple key processes including appetite, body temperature, blood clotting, energy expenditure, glucose homeostasis, inflammation, insulin sensitivity, reproduction, ageing and cancer (Bravo-Sagua et al., 2016), increasing studies begin to explore the functional roles of these circulating adipocyte-secreted factors in human cancer. To date, diverse adipokines such as adiponectin, leptin, resistin, plasminogen activator inhibitor-1 (PAI-1), vascular endothelial growth factor (VEGF), TNF-α, IL-6, autotaxin, fatty acid-binding proteins (FABPs) are being extensively investigated (Guaita-Esteruelas et al., 2017). Among them, adipose-derived FABP4 and FABP5 are identified as critical proteins particularly in lipid-related metabolic processes upon their overexpression in human malignancies including breast, prostate, colorectal, and ovarian cancers. For instance, CAAs interact metabolically with cancer cells through fatty acid transport via FABP4 in ovarian cancer, while FABP4 is involved in lipid transfer between adipocytes and cancer cells, a process that engages the fatty acid oxidation pathway to aid in cancer progression (Nieman et al., 2013). Interestingly, FABP4 knockdown in endothelial cells causes increased fatty acid oxidation and reactive oxygen species (ROS) generation, but decreased angiogenesis, growth, and metastasis in ovarian tumor xenografts (Harjes et al., 2017). Usually, high FABP4 expression levels in human primary tumors are correlated with elevated incidence of residual disease relapse after primary debulking surgery of HGSC, raising the possibility of exploring FABP4 as a candidate biomarker of residual disease in this condition (Tucker et al., 2014).

A recent *in vivo* time-course study disclosed the inverse relationship between metastatic rate and omental adipocyte content, while both milky spots and adipose tissues have specific roles in colonization of the omentum by ovarian cancer cells (Clark et al., 2013). Due to physical proximity to the primary abdominal malignancies, adipose tissues from the peritoneal cavity are frequently observed as a metastasis site for colorectal, gastric, pancreatic, uterine, and ovarian cancers (Guaita-Esteruelas et al., 2017). Obesity contributes to ovarian cancer metastasis, and there is a negative correlation between obesity and survival of ovarian cancer patients (Liu et al., 2015). Ovarian cancer cells that have metastasized to adjacent adipose tissues have upregulated expression of genes encoding fatty acid transport proteins such as CD36 and FABP4, together with other molecules including CD31, CD34, VEGFR1, and VEGFR2 (Gusky et al., 2016). Unlike many solid malignancies, HGSC rarely metastasizes outside the adipocyte-rich abdominal cavity (Kobayashi et al., 2017). A recent study discovered that salt-inducible kinase 2 (SIK2) overexpressed in adipocyte-rich metastatic deposits promotes abdominal metastasis, while adipocyte-induced calcium-dependent activation and autophosphorylation of SIK2 not only augments adenosine monophosphate-activated protein kinase (AMPK)-induced acetyl-CoA carboxylase phosphorylation but also activate the PI3K/AKT pathway via p85a-S154 phosphorylation (Miranda et al., 2016). The data identified SIK2 as a key molecule at the hub of the adipocyte-induced signaling cascades and provided a strong rationale for targeting SIK2 in ovarian cancer therapy.

## TUMOR-ASSOCIATED MACROPHAGES

Although recruitment of tumor-infiltrating leukocytes is one of the natural immune responses to tumorigenesis, numerous studies have identified that distinct leukocyte populations except lymphocytes, are indeed tumor promoting rather than tumor suppressing (Lu et al., 2017). Lymphocytic infiltration is associated with favorable prognosis and improved rates of both progression-free and overall survival of EOC patients (Sato et al., 2005b; Zhang et al., 2003). In particular, the presence of lymphocyte markers including CD20, FoxP3, and T cell intracellular antigen-1 (TIA-1) are indicators of positive prognosis for patients manifesting HGSC (Milne et al., 2009), indicating recruitment of distinct populations of T cells to the tumor site to generate cytotoxic effects. Despite the presence of immune-associated anticancer activities in the TME, most cancer cells can manage to escape immune surveillance. For example, Hospicells expressing the cell surface markers CD9, CD10, CD29, CD146, and CD16 in stroma produce a large amount of nitrous oxide (NO), which suppresses CD4(+), CD8(+) and Vγ9Vδ2 T cell proliferation and cytokine production, eventually conferring chemoresistance on ovarian cancer cells (Martinet et al., 2010). Thus, targeting Hospicells can be an alternative approach to strengthen the efficacy of chemotherapy via recovery of immune responses against cancer cells.

Cancer cells and macrophages have bidirectional interactions through the exchange of soluble factors, a process that remarkably influences the behavior and phenotype of both cell populations. By exploring the contribution of stromal cell-released MMP9 to ovarian tumor growth, a former study showed that cancer cells can change macrophage secretion of cytokines, chemokines, MMPs to enhance tumor growth in peritoneal cavities of nude mice (Huang et al., 2002). As supporting evidence, M2 macrophage (or tumor-associated macrophage, TAM)-secreted MMP9 promotes release of membrane-associated heparin-bound epidermal growth factor (HB-EGF) from the same cells, a process that increases expression of MMP9 in OVCA433 ovarian cancer cells, generating a positive feedback loop to drive growth factor release and accelerate proliferation via co-culture (Carroll et al., 2016). Compared with MMP9(+/+) mice, MMP9(−/−) animals implanted with human ovarian cancer cells have decreased microvessel density and macrophage infiltration into the lesions (Huang et al., 2002). As supporting evidence, upon co-culture of TAMs and ovarian cancer cell line SKOV3, TAMs significantly upregulated MMP2, MMP9, and MMP10 expression and enhanced SKOV3 cell invasion via TLRs signaling pathway, validating the positive association of TAMs with metastasis and advancement of human ovarian cancer (Ke et al., 2016).

In addition, ovarian cancer cells induce dynamic changes in macrophage cytokine, chemokine, and MMP mRNA, and protein-inducing mediators observed in human cancer patients, forming a phenotype similar to that of TAM (Hagemann et al., 2006). Additionally, scavenger receptor expression is also reduced in TAM from ovarian tumors treated with TNF-α antibodies or developed in TNF-α(−/−) mice, indicating chemical communication between cancer cells and macrophages as an important factor in regulating the local microenvironment (Hagemann et al., 2006). A transcriptome-wide global map of signaling pathways in the ovarian TME associated with clinical outcome was recently established via investigation of cancer cell- and TAM-specific transcriptomes from ovarian cancer ascites. STAT3-inducing cytokines, fibroblast growth factor (FGF), and WNT signaling pathway components, semaphorin axon and ephrin guidance proteins, and BMP/TGF-β-activated axis, are evidently associated with early relapse (Reinartz et al., 2016). In addition, TAM-derived phospholipase A2 group VII (PLA2G7) and its product arachidonic acid (AA), together with the AA metabolites including prostaglandin E2 (PGE2), prostaglandin I receptor (PGI2), and leukotriene B4 (LTB4)-associated signaling pathways are also correlated with disease recurrence, indicating a signaling network that operates in the ovarian TME with previously unknown pathway constituents of clinical relevance (Reinartz et al., 2016).

Polarization of macrophages and monocytes toward an M2 phenotype can be promoted by coagulation factor XII (FXII), thrombin, leukemia inhibitory factor (LIF), IL-6 or colony stimulating factor-1 (CSF1), each creating an immune-deficient microenvironment (Alvero et al., 2012; Duluc et al., 2007; Wang et al., 2010; Zhang et al., 2010). Although TAM interferes with normal T lymphocyte generation and function, contributing to tumor tolerance as a tumor-trophic cell lineage, some immunological factors are able to reverse these TAM-specific immunosuppressive properties. In the presence of ovarian ascites, IFNγ skews monocyte differentiation from TAM and turn them into M1-polarized immune-competent macrophages, suggesting that IFNγ overrides TAM-induced immunosuppression by preventing TAM generation and that IFNγ holds the potential to enhance the effectiveness of anticancer immunotherapies by promoting the expansion of effector T cells (Duluc et al., 2009).

Rat IgE and IgG monoclonal antibodies specific for the folate receptor (FRα), a protein expressed on human ovarian cancer cells, can markedly reduce lung metastases (Josephs et al., 2017). This change is accompanied by increased TNF-α^+^ and CD80^+^ macrophage infiltration, together with elevated TNF-α and the monocyte chemotactic protein 1 (MCP-1, or CCL2) in bronchoalveolar lavage fluid of the lung, suggesting anticancer IgE reprograms macrophages in the TME (Josephs et al., 2017).

Although there are similarities between TAMs from human ovarian carcinoma ascites, monocyte-derived macrophages (MDMs) and resident peritoneal macrophages (pMPHs), a recent study identified a TAM-specific signature of 30 genes, which are upregulated as opposed to both MDMs and pMPHs and associated with ECM remodeling, indicating a distinct role for TAMs in ovarian cancer invasion and metastasis (Finkernagel et al., 2016).

A recent study explored the relationship between cancer cell-secreted exosomes and TAM polarization under hypoxic conditions, and found hypoxia increases microRNA-940 (miR-940) expression in exosomes derived from EOC cells (Chen et al., 2017). Interestingly, uptake of exosome-delivered miR-940 in macrophages stimulated an M2 polarization, while the M2 subtype macrophages (TAMs) further enhanced EOC proliferation and migration. Furthermore, TAM-derived exosomes can target the miR-146b-5p/TRAF6/NF-κB/MMP2 pathway to inhibit endothelial cell migration, a process that is subject to reversal by long non-coding RNAs (lncRNAs) delivered by SKOV3-released exosomes (Wu et al., 2017b).

**Figure 1:**
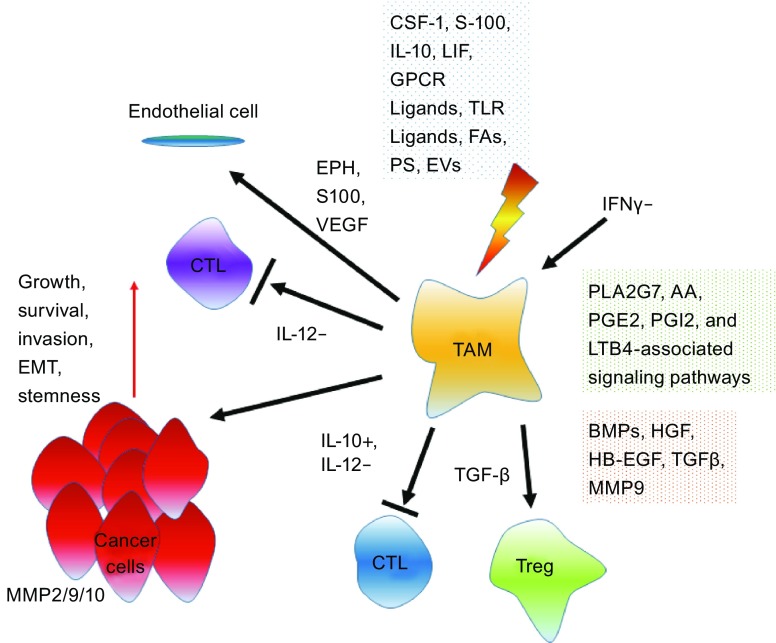
**Functional implications of tumor-associated macrophages (TAMs) in the ovarian cancer microenvironment**. Presence of multiple mediators in the TME and lack of interferon γ (IFNγ) allow activation of TAMs. In response to various stimuli, TAMs release diverse soluble factors that impinge on ovarian cancer cells and resident host cells while minimizing the expression of essential anticancer factors such as IL-12. CSF1, colony stimulating factor 1; LIF, leukemia inhibitory factor; GPCR, G protein coupled receptor; TLR, toll-like receptor; FA, fatty acid; PS, phosphatidylserine (PS); EV, extracellular vesicle; AA, arachidonic acid; PGE2, prostaglandin E2; PLA2G7, phospholipase A2 group VII; PGI2, prostaglandin I receptor; LTB4, leukotriene B4; BMP, bone morphogenetic protein; HGF, hepatocyte growth factor; HB-EGF, heparin-bound epidermal growth factor; TGF-β, transforming growth factor β; MMP, matrix metalloproteinase; EMT, epithelial–mesenchymal transition; CTL, cytotoxic T lymphocyte; Treg, regulatory T cell; EPH, ephrin; S100, S100 calcium binding protein; VEGF, vascular endothelial growth factor; IL-12−, IL-12 reducing; IL-10+, IL-10 enhancing

To date, many efforts are made to target TAMs, and some have shown promising potential in anticancer regimens. Synthesis of tumor-promoting factors including CCL2 and IL-6 is minimized in TAMs and ovarian cancer cells upon treatment by Trabectedin (Yondelis), an agent that originates from the tunicate Ecteinascidia turbinate, binds DNA minor grooves and inhibits monocyte differentiation into macrophage (Allavena et al., 2005). The inhibitory effect of Trabectedin on macrophage differentiation, viability, and cytokine production contributes to its anticancer activity in inflammation-associated human tumors. A phase II study on 147 patients with ovarian cancer who had the experience of platinum-containing chemotherapy, consolidated Trabectedin as an active agent in ovarian cancer clinics (Musrap and Diamandis, 2012; Simpson, 2007) (Table 1). However, the vast majority of these patients reported toxic effects, including fatigue, granulocytopenia, nausea and vomiting, suggesting that definition of a possible role for this drug requires continued investigation (Fig. 1).

**Table 1:** Therapeutic agents that target the tumor microenvironment in clinical trials

Drug/Agent	Type	Target/Mechanism	Impact on the TME	Stage in clinical trials
Infliximab	Monoclonal antibody	Binds to TNF-α with high affinity	Decreases levels of pro-inflammatory cytokines	Phase 1
Etanercept	p75 TNF receptor fusion protein	TNF-α blocker	Inhibits actions of TNF-α	Phase 2
Siltuximab	Monoclonal antibody	Neutralizes IL-6	Inhibits functional activity of IL-6	Phase 3
Trabectedin	Tetrahydro-isoquinoline alkaloid	Binds minor groove of DNA, preventing cell cycle completion; causes apoptosis	Inhibits monocyte-to-macrophage differentiation; decreases production of pro-tumoral cytokines	Phase 3
Sibrotuzumab	Monoclonal antibody	Binds to FAP	Targets major constituents of tumor stroma	Phase 2
Volociximab	Monoclonal antibody	Binds α5β1 integrins	Blocks cancer cell attachment to mesothelium	Phase 2
Bevacizumab	Monoclonal antibody	Binds all isoforms of VEGFA	Suppresses angiogenesis	Phase 3

Representative therapeutic agents that target specific components of the TME and are currently in cancer clinical trials. Data adapted from Musrap and Diamandis with permission from Molecular Cancer Research, copyright 2012, with agent stage-relevant information updated to the date of publication.

## OTHER INNATE IMMUNE CELLS

In addition to TAMs, other innate immune cell subsets are also essential and can generate efficient but mechanistically distinct tumor-associated influences on the development of ovarian cancer. A former study indicated that the tumor-associated inflammatory mediator PGE2 attracts myeloid derived suppressor cells (MDSCs) into ascites of ovarian cancer patients, a process dependent on the expression of C-X-C chemokine receptor type 4 (CXCR4) and its ligand CXCL12 in the ascites microenvironment (Obermajer et al., 2011). The study revealed that frequencies of CD11b(+)CD14(+)CD33(+)CXCR4(+) MDSC migration closely correlates with PGE2 and CXCL12 levels in patient ascites, suggesting a central role for PGE2 in MDSC accumulation regulated by the CXCL12/CXCR4 pathway and providing a novel rationale to target PGE2 signaling in ovarian cancer treatment (Obermajer et al., 2011).

Moreover, MDSCs produce a substantial amount of IL-10, which allows to form an immunosuppressive *in vivo* niche, implying the critical role of IL-10 for development of a tumor-permissive TME (Hart et al., 2011). Further, IL-10 plays a unique role that is not redundant with those of other immunosuppressive molecules, as IL-10 signaling blockade alleviates MDSC-mediated immunosuppression and improves survival, defining IL-10 as a fundamental immune cell modulator in the ovarian TME and represents a viable target for therapeutic strategies (Hart et al., 2011). Although the molecular pathways associated with the differentiation and function of MDSCs in tumorigenesis have been reported (Condamine et al., 2015), the specific mechanisms that recruit MDSCs to the primary tissue and ovarian cancer ascites remain largely unclear.

Natural killer (NK) cells compose another important subset of innate immune cells that actively recognize and eliminate cancer cells. CD16 receptor, NKG2D receptor and natural cytotoxicity receptors, mediate NK-dependent immune surveillance in the TME. In ovarian cancer ascites, two main pathways that suppress NKG2D and NKp30 activity have been recognized. Specifically, migration inhibitory factor (MIF) promotes the immune escape of ovarian cancer cells by transcriptionally down-regulating NKG2D *in vitro* and *in vivo*, thus impairing NK cell cytotoxicity towards neoplastic cells (Krockenberger et al., 2008). Enhanced expression of B7-H6, the recently identified B7 family member as a ligand for NKp30 on NK cells, is positively correlated with ovarian cancer metastasis (Zhou et al., 2015). However, NK cells from patients of low NKp30 expression display decreased IFNγ production and reduced cytolytic function in targeting cells that express surface B7-H6, suggesting a novel mechanism that allows the TME to promote cancer cell escape from immune surveillance (Pesce et al., 2015). A recent study reported the functional difference between NK cells in EOC cell-free ascites and those in ascites with EOC cells, while impaired NK cell response to IL-2 in ascites containing EOC cells is indicative of an immunosuppressive TME (da Silva et al., 2017).

Upon exposure to either inflammatory factors including IFNα, IL-15, IL-12 and IL-2, or ovarian cancer cells, NK cells primed by IL-18 can release chemokines CCL3 and CCL4 to attract immature dendritic cells (iDCs) (Wong et al., 2013). Crosstalk between NK and DC via a CCR5-dependent mechanism causes upregulation of CCR5 and CXCR3 ligands (CCL5, CXCL9, and CXCL10) on DC cells, stimulating recruitment of type-1 effector CD8(+) T (Teff) effector T cells to the TME (Wong et al., 2013). Thus, NK cell activation is critical for elimination of target cancer cells and development of an anticancer immune response. As an interesting point, an IL-15 super-agonist complex ALT-803ALT-803 enhances NK cell cytotoxicity against ovarian cancer and can rescue functionality of NK cells isolated from ovarian cancer patient ascites, suggesting that ALT-803 has the potential to enhance NK cell-based immunotherapeutic efficiency for ovarian cancer treatment (Felices et al., 2017).

## T LYMPHOCYTES

Enhanced infiltration of tumor-infiltrating lymphocytes (TILs) is associated with increased levels of cytokine IFNγ, a prognostic factor that indicates better survival for ovarian cancer patients (Zhang et al., 2003). Specifically, high frequency of intraepithelial CD8^+^ T cells expressing the αE integrin subunit CD103, which may serve as a novel marker for enriching the most beneficial subsets of TILs for immunotherapy, correlates with enhanced survival in ovarian cancer, suggesting that CD8^+^ T lymphocytes directly contribute to the anticancer effects (Komdeur et al., 2016; Sato et al., 2005b; Webb et al., 2014). However, CD4^+^ T cells minimized the beneficial effects of CD8^+^ T cells through CD25(+) forkhead box P3 (FOXP3)(+) regulatory T cells (Treg, suppressor T cells), as evidenced by the significantly shortened median survival of patient subgroups that had high CD4^+^ versus CD8^+^ T cell ratios (Sato et al., 2005a). Data from combined cytokine studies, neutralization experiments, and proliferation assays unveiled that induction of CD8(+) Tregs under *in vitro* conditions relies at least partially on TGF-β1 to exert their suppressive function, a process that is critically mediated by activation of p38MAPK, suggesting p38MAPK as a potential therapeutic target in ovarian cancer immunotherapy (Wu et al., 2016a).

Beyond controlling autoimmunity, allergy, and inflammation in mammals, Tregs are responsible for the maintenance of immune homeostasis in the course of host response to tumorigenesis (Geis et al., 2015). Specifically, Tregs inhibit the proliferation of CD8^+^ T cells as well as their production of IFNγ and IL-2, counteract the protective effect of cytotoxic T cells in ovarian cancer tissues, thus reversely correlated with patient survival (Curiel et al., 2004). Moreover, the ovarian cancer microenvironment can induce migration of CTLA4^+^ FOXP3^+^ GITR^+^ Tregs via the chemokine CCL22 and its receptor CCR4, the latter expressed by infiltrating Tregs (Curiel et al., 2004; Landskron et al., 2015). Importantly, Tregs in the malignant ascites are more activated and proliferate faster than blood-derived cells from the same patient, while the Treg number in patient ascites positively correlates with the epithelial cell number in effusion (Landskron et al., 2015). Interestingly, there is interaction between Treg cells and TAMs in the TME of epithelial ovarian cancer, as is evidenced by a recent study reporting that IL-10 released by TAMs enhances the frequency of Treg cells via activation of Foxp3 during T-cell differentiation and accelerates disease progression (Zhu et al., 2016).

As Tregs suppress tumor-specific T cell immunity and contribute to cancer cell expansion, a strategy exploited by cancer cells to escape immune surveillance, interfering with Treg migration or their function may help prevent disease from further exacerbation. For instance, an anti-CCR4 antibody mAb2-3 blocks Treg migration and stimulates IFNγ secretion by CD8^+^ T cells, eventually enhancing anticancer response in an experimental model bearing CCL22-secreting ovarian cancer cells (Chang et al., 2016). As mAb2-3 induces CD25 shedding from Tregs and causes decreased IL-2-dependent survival of these animals, mAb2-3 represents a promising agonist antibody to restore anticancer immunity by modulating Treg activity (Chang et al., 2016). Interestingly, CD8^+^ Treg cells co-cultured with SKOV3 have lower glycolysis-associated gene expression than CD8^+^ T cells cultured alone (Wu et al., 2016b). As the glycolysis-related gene expression pattern is also minimized in the CD8^+^ T cells of ovarian cancer patients, tumor-stroma mutually remodeled metabolic processes may be a potential mechanism for CD8^+^ Treg induction and reprogramming (Wu et al., 2016b).

**Figure 2:**
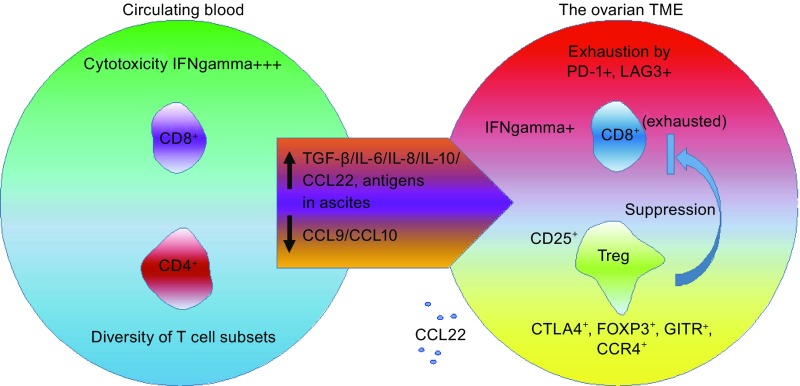
**The balance between CD8** ^**+**^ **T cell and Treg cells in the ovarian cancer microenvironment**. Signals provided by cytokines present in ovarian cancer ascites (IL-6, IL-10) and by dendritic cells induce exhaustion of CD8^+^ T cells. Cells co-expressing the inhibitory receptors, programmed cell death protein 1 (PD-1) and lymphocyte activation gene 3 (LAG3), exhibit significant impairment in the production of interferon γ (IFNγ) and tumor necrosis factor α (TNF-α). Specifically, high antigen concentrations in the ascites can induce CD8^+^ T-cell exhaustion. Moreover, impaired expression of the T helper 1-associated chemokines CCL9 and CCL10 weaken the migration of CD8^+^ T cells into the TME. However, the preferential migration of immunosuppressive FOXP3^+^CTLA4^+^, GITR^+^CCR4^+^ Tregs is stimulated by the chemokine CCL22. CD4^+^, CD4 positive; CD8^+^, CD8 positive; CD25^+^, CD25 positive; CTLA4^+^, cytotoxic T-lymphocyte associated protein 4 positive; Treg, regulatory T cell; GITR^+^, TNF receptor superfamily member 18 positive; FOXP3^+^, forkhead box P3 positive; IL-6, interleukin 6; IL-8, interleukin 8; IL-10, interleukin 10; CCR4^+^, C-C motif chemokine receptor 4 positive; CCL9, C-C motif chemokine ligand 9; CCL10, C-C motif chemokine ligand 10; CCL22, C-C motif chemokine ligand 22

Malignant ascites represents a proinflammatory and immunosuppressive reservoir enriched in chemokines, cytokines, growth factors, and immune cells which together form a pro-tumorigenic microenvironment that allows tumor cell growth and confers resistance to standard treatments (da Silva et al., 2017). However, a new study disclosed that co-treatment with dabigatran etexilate, a direct thrombin inhibitor, significantly promoted the genotoxic activity of cisplatin during ovarian cancer progression via alleviation of the immunosuppressive microenvironment, suggesting that thrombin may be a candidate therapeutic target for ovarian cancer patients (Alexander et al., 2016). Another study explored the effect of neoadjuvant chemotherapy (NACT) on immune activation in stage IIIC/IV tubo-ovarian HGSC, and found that NACT enhanced host immune response but can be interfered by high levels of PD-1, PD-L1 and CTLA4, implying sequential chemoimmunotherapy may provide therapeutic benefits in advanced HGSC populations (Bohm et al., 2016). Data from clinical investigation of ovarian cancer-associated CD4^+^CD25^+^, CD4^+^CD25^+^Foxp3^+^, CD8^+^CD28^−^, and CD8^+^Foxp3^+^ Tregs and their postoperative alterations indicated that Tregs percentage continues to decline in postoperative period with remarkable correlation with the tumor burden, thus can be used as an important factor in monitoring the immunological status of ovarian cancer patients (Wu et al., 2017a) (Fig. 2).

## ANGIOGENESIS AND NEOVASCULATURE

In the TME, cancer cells rely on a constant supply of nutrients and oxygen, which is supported by the formation of new blood vessels. Several pathways are involved in the regulation of the growth and maintenance of neovasculature, a process that can be mediated by proangiogenic factors secreted by both tumor and stromal cells (Saharinen et al., 2011). MMP1-mediated activation of the G protein coupled receptor (GPCR) protease-activated receptor-1 (PAR1) stimulates ovarian cancer cells to release CCL2, IL-8, growth regulated oncogene-a (GROa), chemokines that induce endothelial cell proliferation, tube formation, angiogenesis, and metastasis in peritoneal mouse models of ovarian cancer (Agarwal et al., 2010).

A new study examined VEGF expression in benign, borderline, and malignant neoplasms to correlate it with histological grade and stage of ovarian cancer patients. Despite notable VEGF expression in some benign and borderline neoplasms, high VEGF expression was mostly observed in carcinomas, suggesting EOC as a candidate for VEGF-targeting therapy (Mukherjee et al., 2017). Although the globally commercialized VEGF-specific antibody, Avastin, displayed effectiveness in suppressing angiogenesis in these animals, IL-8 and GROa-dependent endothelial tube formation *in vitro* remained unchanged (Agarwal et al., 2010). Another study identified tumor necrosis factor superfamily-15 (TNFSF15), an endogenous suppressor of neovascularization, plays a critical role in the physiologically normal ovary but is lost in ovarian cancer (Deng et al., 2012). TNFSF15 silencing before and after inoculation of ovarian cancer ID8 cells to mice markedly increases angiogenesis and tumor growth, suggesting downregulation of TNFSF15 by cancer cells and tumor infiltrating macrophages (TIMs) and lymphocytes is a pre-requisite for neovascularization in ovarian cancer (Deng et al., 2012).

**Figure 3:**
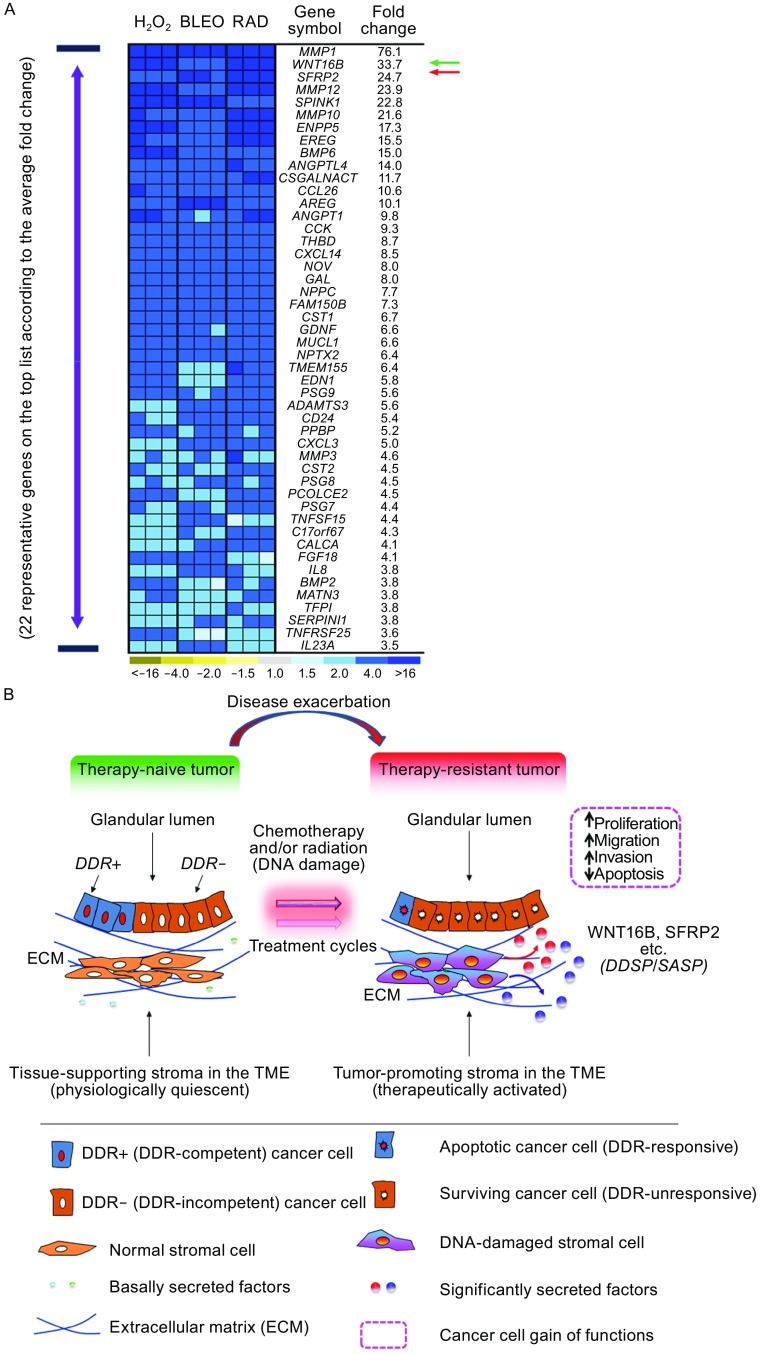
**WNT16B and SFRP2 are significantly produced in human stromal cells during chemotherapy or radiation, and promote therapeutic resistance to surviving cancer cells**. (A) Genome-wide expression pattern of primary normal human stromal cells. Heatmap depicts the relative mRNA abundance after exposure of cells to typical DNA damaging agents (H_2_O_2_, hydrogen peroxide; Bleo, bleomycin; Rad, ionizing radiation). (B) Working model for cancer cell non-autonomous therapeutic resistance acquired from the TME upon anticancer treatments particularly genotoxic chemotherapy and radiation. Therapeutic agents cause apoptosis in subsets of cancer cells by eliciting a DDR, while cancer cells with DDR deficiency (DDR-insensitive, or DDR-) escape from cytotoxic attack. Simultaneously, senescence is induced in stromal cells adjacent to epithelial cells surrounding the gland, with a secretory phenotype DDSP developed after DDR events. A persistently activated signaling network is triggered by the DNA strand breaks. The DDSP is usually characterized by a spectrum of autocrine- and paracrine-acting proteins. The soluble factors reinforce the senescent phenotype in damaged cells, enhance cancer cell repopulation, with increased occurrence of tumor relapse and distant metastasis. As exemplified by the recently reported WNT16B, a handful of co-synthesized factors including SFRP2 hold the potential to serve as both a serum biomarker to determine treatment index, and a therapeutic target to minimize the TME-conferred therapeutic resistance. DDR, DNA damage response; ECM, extracellular matrix; TME, tumor microenvironment. Color images of (A) adapted from Sun et al. with permission from Nature Medicine, copyright 2012

Pericytes, endothelial, and smooth muscle cells are stromal components that play an essential role in angiogenesis (Bussard et al., 2016; Huijbers et al., 2016). Although pro-angiogenic factors are produced by stromal and epithelial cells during early carcinogenesis or as a consequence of inflammation, enhanced angiogenesis is usually a late event in cancer progression (Hanahan and Weinberg, 2011). Upon treatments by anticancer agents, stromal expression of VEGF and other angiogeneic factors including angiopoietin 1 (ANGPT1) and angiopoietin-like 4 (ANGPTL4) is increased, functionally stimulating vasculature development within the drug-remodeled TME (Coppe et al., 2008; Sun et al., 2012). Synthesis of the secreted frizzled-related protein 2 (SFRP2), a canonical modulator of Wnt signaling, is enhanced in damaged prostate stroma by chemotherapeutics, which promotes angiogenesis via a calcineurin/NFAT pathway in a non-canonical manner (Courtwright et al., 2009; Siamakpour-Reihani et al., 2011). Of note, SFRP2 acts as an agonist of WNT16B, another extracellular factor released by the therapy-damaged TME which significantly promotes drug resistance of residual cancer cells in human prostate, breast, and ovarian malignancies in post-treatment stage (Sun et al., 2012; Sun et al., 2016) (Fig. 3). Increasing interest has emerged in targeting angiopoietin growth factors (mainly Ang1, Ang2, Ang4) that promote CAF accumulation and tumor angiogenesis in the TME, while Tie1 and Tie2 (also as TEK) receptors that regulate the maturation and plasticity of blood vessels are also considered as valuable targets (Augustin et al., 2009; Brunckhorst et al., 2014). Anti-angiogenic therapies targeting tumor vasculature represent a practical approach upon combination with other conventional treatments in ovarian cancer management. Minimizing angiogenesis in cancer patients to overcome side effects caused by cytotoxicity is thus a promising strategy to block neoplastic growth and deprive cancer cells of microenvironment-conferred malignancy.

Continuous accumulation of ascites, chronic inflammation, and elevated VEGF concentrations are among the typical hallmarks of ovarian cancer progression. Overexpressed c-myc remarkably enhances VEGF concentrations in ascites of ovarian cancer mice, while transduced Kras significantly enhances inflammatory cytokine concentrations and increases the number of neutrophils in animal ascites, suggesting that oncogenes can favor disease progression by modulating the ovarian TME (Yoshida et al., 2016). Moreover, peritoneal cavity and retroperitoneal lymph node represent main routes for EOC dissemination, while VEGF-mediated angiogenesis is an important mechanism that promotes ovarian cancer progression. As patients with high VEGFC levels display a significantly worse overall survival than those with low VEGFC expression, VEGFC may serve as a clinical marker to identify patients of increased risk for lymphatic metastases, a subpopulation that might benefit from VEGFC-specific treatment regimens (Kuerti et al., 2017).

## OVARIAN CANCER-ASSOCIATED EVS

In the course of ovarian cancer progression, intercellular communication defines the pace of neoplastic cell survival and expansion. EVs are released by almost all cell types in the TME, and mediate the transfer of proteins, lipids, and nucleic acids (DNAs, mRNAs, miRNAs, and lncRNAs) between or within tumor and stroma (Han et al., 2017). EVs can be subdivided into exosomes (30–150 nm) and microvesicles (100 nm–1 μm), depending on whether they are originated from multivesicular bodies (MVBs) or shed from the plasma membrane (Budnik et al., 2016). Stroma-released EVs can modulate the cancer cell invasion and metastasis, while cancer cells also generated EVs to induce functional transition of nearby stromal cells to favor disease progression. EV formation and release are regulated by multiple factors, while either endogenous or exogenous factors can change the number, content and type of EVs, thereby substantially altering their activities. For instance, biogenesis and release of cancer exosomes are regulated by endosomal sorting complex required for transport (ESCRT), intracellular calcium levels and structural scaffolding, and subject to stimulation by stresses such as microbial attacks (Azmi et al., 2013). Signal transduction via EVs adds another level of complexity to the cell communication network as EVs simultaneously release multiple molecules impinging on signaling pathways in the recipient cell.

As a subtype of non-coding RNAs, miRNAs control expression of target genes posttranscriptionally, while dysregulation of miRNAs is involved in multiple steps of ovarian cancer development. Exosomal miRNAs isolated from the serum or ascites have potential clinical values for ovarian cancer diagnosis, prognosis, and therapeutics (Nakamura et al., 2016). A former study reported the positivity of 218 out of 467 mature miRNAs isolated from ovarian cancer cells and exosomes of the same patients (Taylor and Gercel-Taylor, 2008). Among them, 8 specific miRNAs had similar levels between cellular and exosomal miRNAs and exhibited correlations in a range of 0.71–0.90. Although EpCAM-positive exosomes were present in both patients with benign ovarian disease and those harboring ovarian cancer, exosomal miRNA from ovarian cancer patients displayed similar profiles and were considerably distinct from those observed in benign cases. Thus, miRNA profiling of circulating cancer exosomes can be potentially employed as a surrogate diagnostic marker for biopsy analysis (Taylor and Gercel-Taylor, 2008). The first study investigating the miRNAs in ovarian cancer tissues and cell lines found that miR-200a, miR-141, miR-200c, and miR-200b were most significantly overexpressed, while miR-199a, miR-140, miR-145, and miR-125b1 were the most downregulated miRNAs (Iorio et al., 2007). In addition, expression of miR-21, miR-203, and miR-205 whose levels were upregulated in ovarian cancer, were significantly elevated in OVCAR3 cells upon treatment by 5-aza-2’-deoxycytidine demethylating, suggesting DNA hypomethylation as a potential mechanism for miRNA overexpression (Iorio et al., 2007). Particularly, the members of miR-200 family maintain epithelial cell integrity by suppression of EMT via direct inhibition of mesenchymal transcription factors zinc finger E-box-binding homeobox 1/2 (ZEB1/ZEB2) and TGF-β, a potent inducer of EMT. Although downregulation of miR-200s in cancer cells promotes EMT and cancer metastasis, these molecules are highly expressed in ovarian cancer and cause metastasis primarily by enhancing cancer cell dissemination within the pelvic cavity (Choi and Ng, 2017).

Increasing lines of evidence suggest that EVs in ovarian cancer ascites are associated with immune suppression, invasion, and treatment resistance (Worzfeld et al., 2017). Ovarian ascites-released EVs reduce the cytotoxicity of peripheral lymphocytes and promote apoptosis of DCs and lymphocytes (Yokoi et al., 2017). Further, EV-associated FAS-L purified from patient ascites triggers FAS-induced apoptosis in a T cell line, highlighting the possibility of elimination of FAS-bearing immune cells including T cells by vesicle-delivered FAS-L, a process that allows immune evasion to support cancer cell survival (Abrahams et al., 2003). EVs from ovarian ascites can also inhibit T cell activation by blocking the T cell signaling cascade, an effect contributed by EV-associated phosphatidylserine (PS) (Kelleher et al., 2015). Depletion of HSP70^+^ EVs and blockade of HSP70/TLR2 interaction with A8, a specific peptide aptamer, can prevent the activation of MDSCs and abolish cancer progression in experimental mice, suggesting the potential of targeting EVs as a novel immunotherapy for ovarian cancer treatment (Gobbo et al., 2016)

Ovarian cancer-derived EVs carry molecules that directly regulate cancer cell migration, including CD24, EpCAM, and soluble activated leukocyte cell adhesion molecule (sALCAM) and soluble L1 (Carbotti et al., 2013; Gutwein et al., 2005; Runz et al., 2007). Malignant ascites-released membrane vesicles contain diverse activated proteases including MMP2, MMP9, uPA and ADAM17/TACE, together promoting ECM degradation and enhancing cancer cell invasiveness and metastasis (Carbotti et al., 2013; Graves et al., 2004). However, EVs may also carry miRNAs that positively or negatively regulate invasion and migration behaviors of cancer cells. For example, miR-6126 is released in a large amount via exosomes from both chemosensitive and chemoresistant ovarian cancer cells, and is correlated with longer overall survival in patients with HGSC (Kanlikilicer et al., 2016). Functionally targeting integrin β1, a key regulator of cancer cell metastasis, miR-6126 acted as a tumor suppressor, and delivery of its mimic to endothelial cells (ECs) generates decreased ovarian cancer cell invasion and migration *in vitro* as well as reduced tumor growth *in vivo* (Kanlikilicer et al., 2016).

Beyond above pathological effects, EV-associated miRNAs can remarkably change the response of recipient cells to various anticancer agents. EV-mediated miR21 transfer from stromal cells including omental CAAs and CAFs to ovarian cancer cells induces resistance to paclitaxel-based chemotherapy by directly targeting the mRNA of apoptotic peptidase activating factor 1 (APAF1), implying inhibition of the stromal-derived miR-21 transfer is an exploitable modality in management of metastatic and recurrent ovarian cancer patients (Au Yeung et al., 2016). A recent study revealed that acquired SMAD4 mutations increase the chemoresistance capacity of EOCs via a novel mechanism through which platinum-resistant cell-derived SMAD4^+^ exosomes perpetuate an EMT phenotype and promote the expansion of platinum-refractory cell subpopulations (Crow et al., 2017). Stable miR-433 expression in A2780 ovarian cancer cells induces a cellular senescence phenotype characterized by typical morphological changes, phosphorylated retinoblastoma (p-Rb) downregulation, and β-galactosidase activity enhancement (Weiner-Gorzel et al., 2015). Further, miR-433-expressing cancer cells release miR-433 into the growth media via exosomes which in turn can generate a senescence bystander effect, thus promoting the potential of recipient cells to survive chemotherapeutic treatment (Weiner-Gorzel et al., 2015). Exosomes released by a cisplatin-resistant derivative of A2780, CP70 cell line, were capable of driving resistance in treatment-naïve A2780 cells (Pink et al., 2015). Similarly, cisplatin-resistant SKOV3 cells can release increased numbers of exosomes, which contain detectable annexin A3, a molecule correlated with platinum resistance of ovarian cancer cells (Yin et al., 2012).

## CONCLUDING REMARKS AND PROSPECTIVE VISTAS

Decades of scientific and clinical efforts in ovarian cancer research provide an important baseline for the development of TME-oriented therapeutic agents that are mainly designed to interfere with tumor–stroma interactions in ovarian cancer patients. To date, many agents have achieved pronounced efficacy in improving patient survival when incorporated into traditional cytotoxic chemotherapies. However, like most human solid malignancies, ovarian cancer has distinct environmental landscapes and display substantial heterogeneity, even standardized treatments elicit a varying degree of responses among patient subgroups. To circumvent the difficulties, emerging new techniques provide substantial benefits for translational research. For instance, engineered nanochips such as Tantalum oxide nanodot arrays of 10–200 nm are now designed as artificial microenvironments, with the experimental setup and methodology specific for appraisal of ovarian cancer invasiveness (Dhawan et al., 2016). This technical advancement presents an optimal diagnostic platform to investigate cancer cell behavior, facilitate “markerless monitoring” of ovarian cancer progressiveness as a pilot model in the transdisciplinary fields of biomedical engineering and cancer research.

There has been a surge in clinical trials with drugs that specifically control transmembrane or cytoplasmic enzymes, induce apoptosis and suppress angiogenesis in site-specific ovarian cancer cells, thus holding considerable promise to design more efficacious protocols. Future exploration in this field should focus on how to improve the practical accuracy of current therapies, to identify an increasing number of ovary-specific molecular targets for optimal effectiveness, and to develop an omics-based system for patient stratification. Altogether, conceptual and technical advancement will allow physicians to make informed decisions in ovarian cancer clinics towards a thorough cure of this gynecological malignancy.
